# High efficacy of tumor-targeting *Salmonella typhimurium* A1-R on a doxorubicin- and dactolisib-resistant follicular dendritic-cell sarcoma in a patient-derived orthotopic xenograft PDOX nude mouse model

**DOI:** 10.18632/oncotarget.8848

**Published:** 2016-04-20

**Authors:** Tasuku Kiyuna, Takashi Murakami, Yasunori Tome, Kei Kawaguchi, Kentaro Igarashi, Yong Zhang, Ming Zhao, Yunfeng Li, Michael Bouvet, Fuminori Kanaya, Arun Singh, Sarah Dry, Fritz C. Eilber, Robert M. Hoffman

**Affiliations:** ^1^ AntiCancer Inc., San Diego, CA, USA; ^2^ Department of Surgery, University of California, San Diego, CA, USA; ^3^ Department of Orthopedic Surgery, Graduate School of Medicine, University of the Ryukyus, Okinawa, Japan; ^4^ Division of Hematology-Oncology, University of California, Los Angeles, CA, USA; ^5^ Department of Pathology, University of California, Los Angeles, CA, USA; ^6^ Division of Surgical Oncology, University of California, Los Angeles, CA, USA; ^7^ PDOX LLC, San Diego, CA, USA

**Keywords:** Salmonella typhimurium A1-R, tumor-targeting, GFP, sarcoma, soft-tissue

## Abstract

Follicular dendritic-cell sarcoma (FDCS) is a rare and recalcitrant disease. In the present study, a patient-derived orthotopic xenograft (PDOX) mouse model of FDCS was established in the biceps muscle of nude mice. The FDCS PDOX was resistant to both doxorubicin (DOX) and NVP-BEZ235, dactolisib (BEZ) an experimental agent which is a dual pan-phosphoinositide 3-kinase-mammalian target of rapamycin inhibitor. However, in contrast to DOX and BEZ, the FDCS PDOX was sensitive to the tumor-targeting bacterial strain, *Salmonella typhimurium* A1-R (*S. typhimurium* A1-R). The combination of *S. typhimurium* A1-R and either DOX or BEZ did not increase the antitumor efficacy of *S. typhimurium* A1-R, indicating that DOX and BEZ were not active in this PDOX model. The efficacy of *S. typhimurium* A1-R in this recalcitrant FDCS gives strong impetus to move bacterial therapy to clinical trials for this disease. The findings of the present study are of particular importance since it demonstrates that *S. typhimurium* A1-R is effective in a PDOX model of FDCS established from a patient who failed DOX therapy.

## INTRODUCTION

Follicular dendritic-cell sarcoma (FDCS) is a highly recalcitrant disease. FDCS is rare and arises from follicular dendritic cells [1]. CHOP chemotherapy, which contains cyclophosphamide (CTX), doxorubicin (DOX), vincristine and prednisone, is most frequently used with FDCS with transient, partial responses observed in some patients [2]. Complete responses to CHOP are infrequent [1]. The 5-year overall survival (OS) for localized FDCS is 55% and for metastatic disease is 38% [3]. Therefore, novel approaches to FDCS are needed [1, 2].

The tumor-targeting *Salmonella typhimurium* A1-R (*S. typhimurium* A1-R) strain was developed by our laboratory [4]. *S. typhimurium* A1-R is auxotrophic for Leu–Arg, which prevents it from mounting a continuous infection in normal tissues. *S. typhimurium* A1-R was able to inhibit or eradicate primary and metastatic tumors as monotherapy in nude mouse models of major cancers [5], including prostate [6, 7], breast [8–10], lung [11, 12], pancreatic [13–17], ovarian [18, 19] stomach [20], and cervical cancer [21], as well as sarcoma cell lines [22–25] and glioma [26, 27], all of which are highly aggressive tumor models.

Previously, we developed a patient-derived nude-mouse model of soft tissue sarcoma resistant to gemcitabine. However, *S. typhimurium* A1-R significantly inhibited tumor growth compared to the untreated mice. These results suggest tumor-targeting *S. typhimurium* A1-R is a promising treatment for chemo-resistant soft tissue sarcoma [28].

Recently, a patient with high-grade undifferentiated pleomorphic soft tissue sarcoma from a striated muscle was grown in the right biceps femoris muscle of mice to establish a patient-derived orthotopic xenograft (PDOX) model. This sarcoma PDOX was sensitive to DOX and *S. typhimurium* A1-R followed by DOX, could eradicate this tumor [25].

The present study evaluates *S. typhimurium* A1-R efficacy on a DOX-resistant FDCS PDOX model established from a patient who failed DOX therapy.

## RESULTS AND DISCUSSION

**Figure 1:**
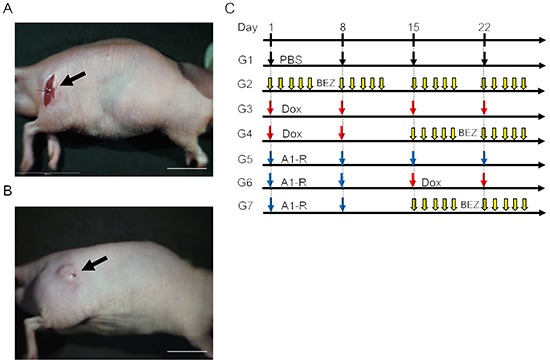
PDOX model of follicular dendritic-cell sarcoma (FDCS) and treatment protocol. **A.** During the sarcoma transplant procedure in the muscle. **B.** Three weeks after implantation. **C.** Treatment protocol.

The treatment schedule for the FDCS PDOX is shown in Figure 1. Three weeks after orthotopic implantation, tumors reached 5 mm in diameter and continued to grow rapidly (Figure 1A).

**Figure 2:**
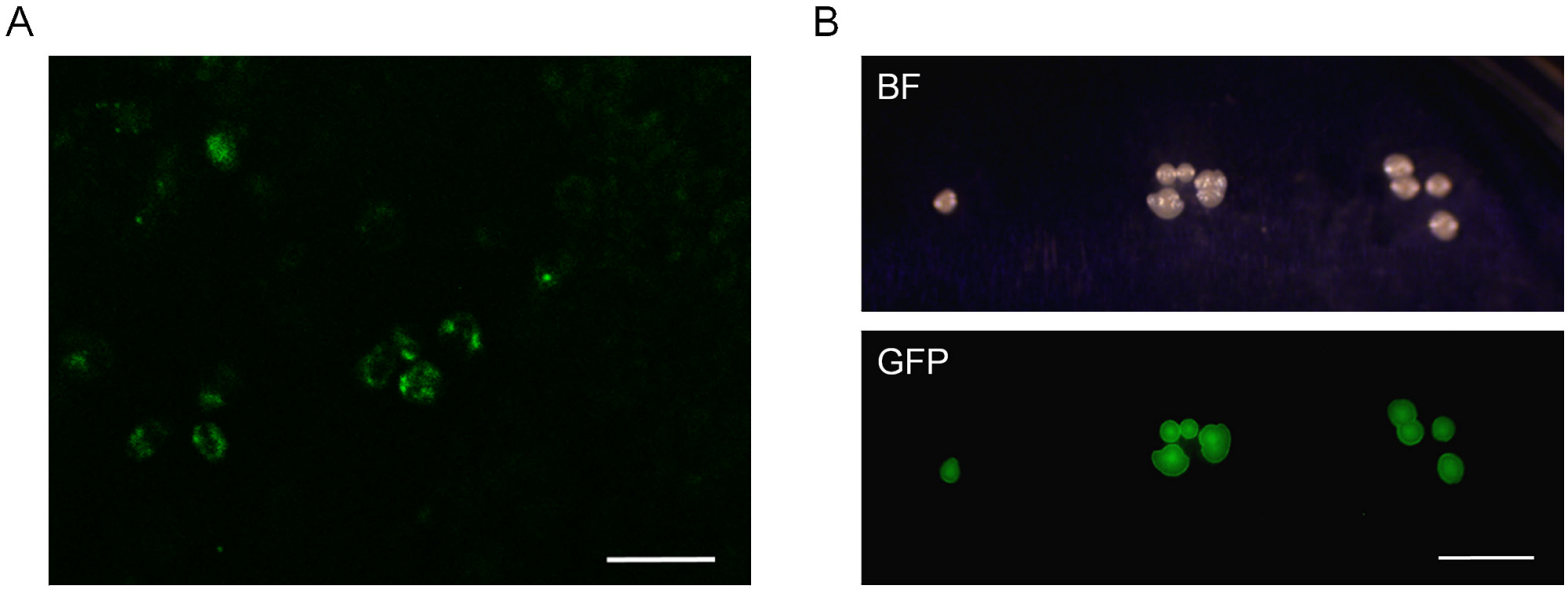
Imaging tumor-targeting *Salmonella typhimurium* A1-R in the FDCS PDOX. **A.** FDCS was resected from a PDOX model after 4 weeks treatment of *S. typhimurium* A1-R and a subsequent two weeks without treatment. FV1000 confocal microscopy. Scale bar = 100 μm. **B.** Colonies of *S. typhimurium* A1-R isolated from the tumor of the bacterially-treated FDCS PDOX after 4 weeks treatment and a subsequent 2 weeks without treatment.

After intraperitoneal (i.p.) administration of *S. typhimurium* A1-R for four weeks, and two subsequent weeks without treatment, the green fluorescent protein (GFP)-expressing bacteria could be visualized by fluorescence imaging in the resected tumor. *S. typhimurium* A1-R was imaged directly as well as by mincing of the tumor and subsequent colony outgrowth from the minced tissue on agar medium (Figure 2).

**Figure 3:**
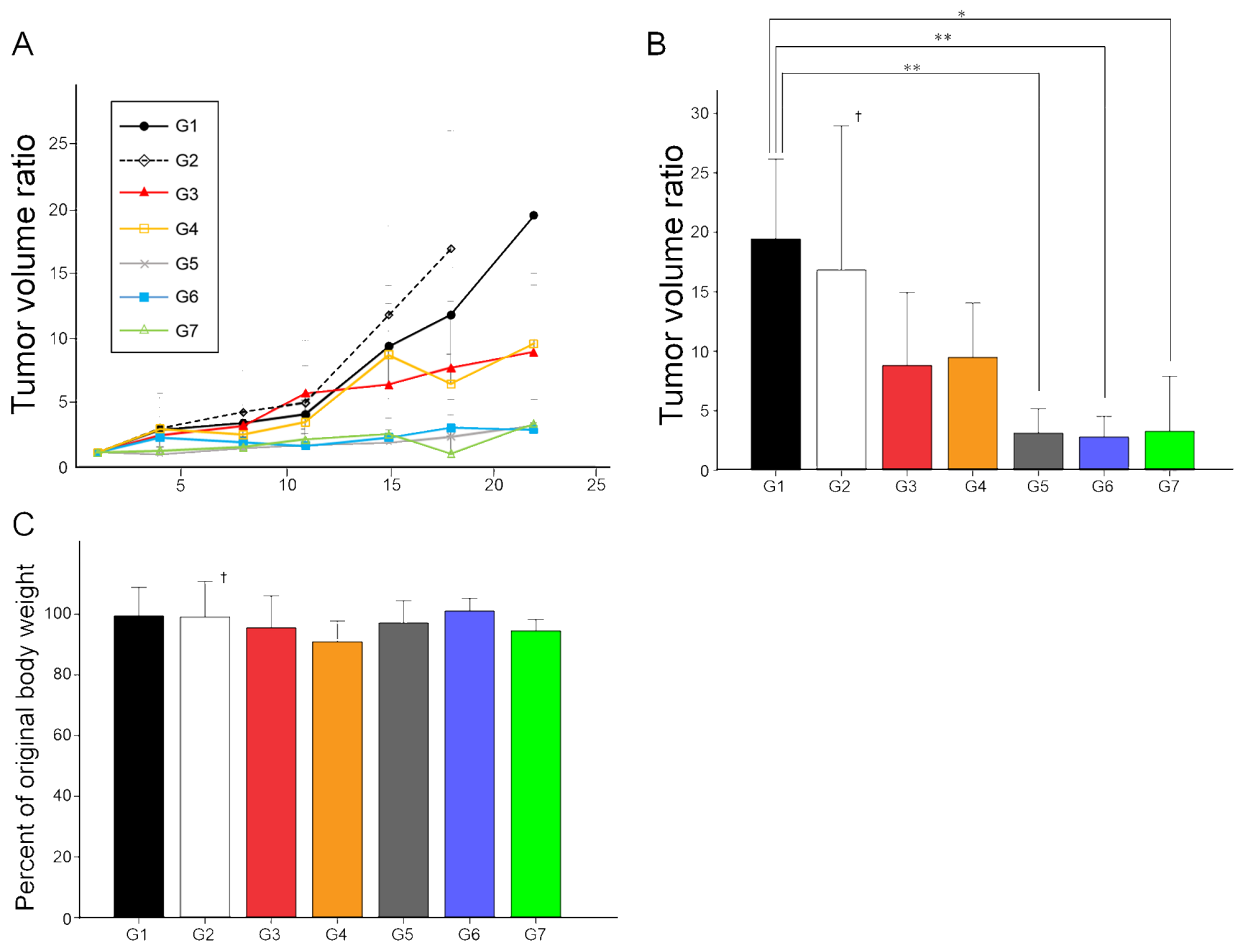
Efficacy of chemotherapy and *S. typhimurium* A1-R in the FDCS PDOX A, B. Group 1, control with PBS, i.p.; Group 2, treated with BEZ, 50 mg/kg oral gavage, q5/W for 4 weeks; Group 3, treated with DOX, 2.4 mg/kg, i.p., qW for 4 weeks; Group 4, treated with DOX, 2.4 mg/kg, i.p., qW for 2 weeks; and BEZ, 50 mg/kg, oral gavage, q5/W for 2 weeks; Group 5, treated with *S. typhimurium* A1-R, 2.5 × 10^7^ CFU, i.p., qW for 4 weeks; Group 6, treated with *S. typhimurium* A1-R, 2.5 × 10^7^ CFU, i.p., qW for 2 weeks followed by DOX, 2.4 mg/kg, i.p., qW for 2 weeks; and Group 7, treated with *S. typhimurium* A1-R, 2.5 × 10^7^ CFU, i.p., qW for 2 weeks followed by BEZ, 50 mg/kg, oral gavage, q5/W for 2 weeks. Tumor volume ratio in Group 5 (3.11 ± 2.05, p < 0.01); Group 6 (2.80 ± 1.72, p < 0.01); and Group 7 (3.28 ± 4.62, p < 0.05) were significantly lower than in Group 1 (19.44 ± 6.70). There were not significant differences between any other groups. **C.** Bar graph shows percentage of original body weight of mice in each group on 22^nd^ day from initial treatment except for Group 2, which was on day-18. Actual weights at these time points were: Group 1: 22.24 ± 1.73; Group 2: 26.13 ± 1.54; Group 3: 22.94 ± 2.01; Group 4: 22.88 ± 1.20; Group 5: 25.32 ± 2.40; Group 6: 25.34 ± 1.52; and Group 7: 23.2 ± 2.21. There were not significant differences between any treated groups and control. *p < 0.05, **p < 0.01. Error bars: ± 1 SD. †The graph of only G2 shows tumor volume ratio on day-18. Error bars: ± 1 SD.

The FDCS PDOX was resistant to doxorubicin (DOX) (*p* = 0.11 at day-22 of treatment, Group 3) (Figure 3). The FDCS PDOX was also resistant to NVP-BEZ235 (dactolisib) (BEZ), which is a dual pan-phosphoinositide 3-kinase-mammalian target of rapamycin mTOR inhibitor [29] (*p* = 0.48 at day-18 of treatment, Group 2). In a Phase II trial, investigators reported a durable partial response in a patient with metastatic FDCS treated with ridaforolimus, an mTOR inhibitor [30]. The FDCS PDOX was also resistant to the combination of DOX and BEZ (*p* = 0.14 at day-22, Group 4).

However, in contrast to DOX and BEZ, the FDCS PDOX was sensitive to the tumor-targeting bacterial strain, *S. typhimurium* A1-R (*p* < 0.05 at day-22, Group 5) (Figure 3A, 3B). The combination of *S. typhimurium* A1-R and either DOX (Group 6) or BEZ (Group 7) did not increase the antitumor efficacy of *S. typhimurium* A1-R (Figure 3), indicating that DOX and BEZ were not active against this tumor. The tumor-volume ratio in Group 5, *S. typhimurium* A1-R (3.11 ± 2.05, *p* < 0.01); Group 6, *S. typhimurium* A1-R and DOX (2.80 ± 1.72, *p* < 0.01); and Group 7, *S. typhimurium* A1-R and BEZ (3.28 ± 4.62, *p* < 0.05) were significantly lower than in Group 1, untreated control (19.44 ± 6.70) (Figure 3B). There were not significant differences between any other groups. Since BEZ alone was inactive, it is not surprising it also had no effect in combination with DOX. Sequential treatment was given with *S. typhimurium* A1-R followed by either DOX or BEZ. The goal of this experiment was to determine if *S. typhimurium* A1-R could sensitize the tumor by decoying the quiescent cells in the tumor to begin to cycle and therefore become more responsive to the chemotherapy [20]. However, the tumor was sufficiently sensitive to *S. typhimurium* A1-R that no further tumor inhibition could be observed in the combination with either an inactive or slightly active drug compared to *S. typhimurium* A1-R alone.

There were no significant body-weight differences between the groups (Figure 3C). Actual mouse weights at day-22 for all groups, except Group 2 which was determined day-18, were Group 1 (untreated control): 22.24 ± 1.73; Group 2 (BEZ): 26.13 ± 1.54; Group 3 (DOX): 22.94 ± 2.01; Group 4 (BEZ + DOX): 22.88 ± 1.20; Group 5 (*S. typhimurium* A1-R): 25.32 ± 2.40; Group 6 (*S. typhimurium* A1-R + DOX): 25.34 ± 1.52; and Group 7 (*S. typhimurium* A1-R + BEZ): 23.2 ± 2.21.

### Histology

**Figure 4:**
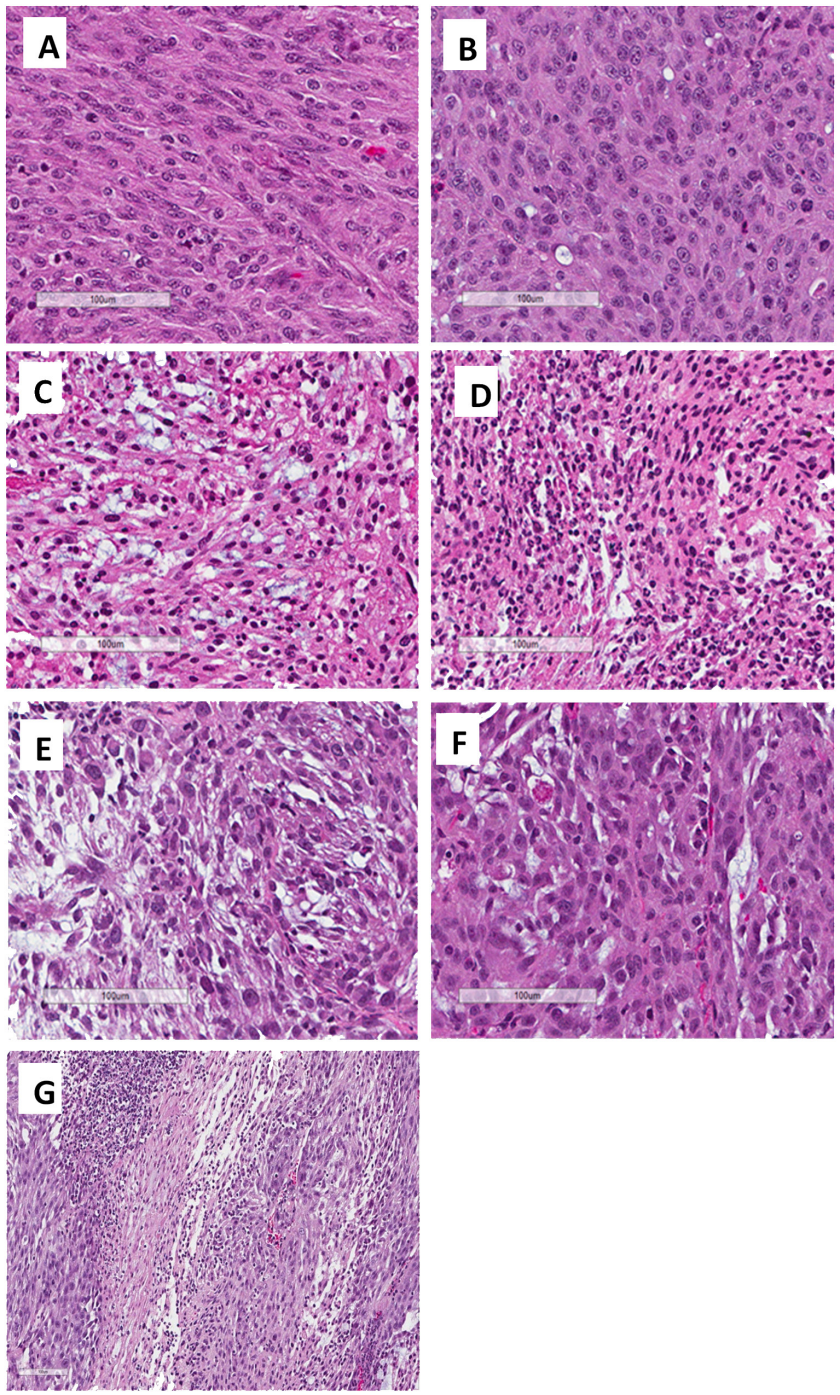
Photomicrographs of H&E-stained slides from the original patient tumor and the PDOX-grown tumor. **A.** The patient's original tumor. **B.** Untreated PDOX tumor. **C.** Treated tumor with BEZ (Group 2, 5% necrosis). **D.** Treated tumor with DOX (Group 3, 20% necrosis). **E.** Treated tumor with *S. typhimurium* A1-R (Group 5, 50% necrosis). **F.** Treated tumor with *S. typhimurium* A1-R and DOX (Group 6, 20% necrosis). **G.** Treated tumor with *S. typhimurium* A1-R and BEZ (Group 7, 60% necrosis). Scale bars: 100 μm.

The patient's original tumor (Figure 4A) and untreated PDOX (Figure 4B) showed identical histologic features which are characteristic for FDCS, including relatively uniform ovoid-to spindle-shaped cells present in short fascicles and storiform patterns with associated lymphocytes sprinkled throughout the tumor. Similar to the original tumor, the PDOX showed a high rate of mitoses (>20/10 High-power fields [HPFs]) (Figure 4B). The patient's original tumor was positive for CD35 and fascin by immunohistochemistry (data not shown), supporting the diagnosis of FDCS.

Review of xenograft sections showed the following approximate amount of necrosis: Group 1: control with PBS (0%, Figure 4B), Group 2: BEZ-235 (5%, Figure 4C), Group 3: DOX (20%, Figure 4D); Group 5: *S. typhimurium* A1-R (50%, Figure 4E); Group 6: *S. typhimurium* A1-R and DOX (20%, Figure 4F); Group 7: *S. typhimurium* A1-R+BEZ (60%, Figure 4G). Group 7 was greatly inhibited by *S. typhimurium* A1-R. Future studies will examine the extent of necrosis in *S. typhimurium* A1-R-treated tumors. The BEZ-treated tumors grew extensively and may have outgrown their blood supply and thus became slightly necrotic.

In a previous study, a human patient with advanced leiomyosarcoma was treated with an intra-tumoral injection of *Clostridium novyi* (*C. novyi*-NT) spores which reduced the tumor within and surrounding the bone [31], indicating the clinical potential of bacterial therapy of sarcoma. Since *S. typhimurium* A1-R is a facultative anaerobe, unlike *C. novyi*-NT which is an obligate anaerobe, it may have more broad application for cancer therapy. *S. typhimurium* A1-R can greatly potentiate cytotoxic chemotherapy [20]. *S. typhimurium* A1-R was recently shown to potentiate DOX in a PDOX model of high-grade undifferentiated soft-tissue sarcoma from a striated muscle [25].

In a Phase I clinical trial of patients with metastatic melanoma and renal carcinoma, the *S. typhimurium* strain tested (VNP20009), attenuated by msbB and purI mutations, was safely administered to patients [32]. The results of the present study suggest *S. typhimurium* A1-R is a candidate for clinical trial to treat DOX-resistant FDCS.

The findings of the present study are of particular importance since it demonstrates that *S. typhimurium* A1-R is effective in the PDOX model of FDCS established from a patient who failed DOX therapy and whose PDOX is DOX-resistant.

Previously developed concepts and strategies of highly selective tumor targeting [33–38] can take advantage of bacterial targeting of tumors, including tissue-selective therapy which focuses on unique properties of normal and tumor tissues [33, 38]. *S. typhimurium* A1-R can possibly overcome de-differentiation of a tumor leading to resistance to targeted chemotherapy, where the targeted protein or pathway may no longer be expressed [38], since *S. typhimurium* A1-R does not depend on such targets [33, 35]. *S. typhimurium* A1-R may also be effectively combined with teratogens which could selectively affect cancer cells that are dedifferentiated [34]. Since *S. typhimurium* A1-R can decoy quiescent cancer cells to begin to cycle, *S. typhimurium* A1-R could be effectively combined with agents that selectively target proliferating cancer cells [36], where normal cells are protected by agents which induce wild type p53 [37].

## MATERIALS AND METHODS

### Mice

Athymic *nu/nu* nude mice (AntiCancer Inc., San Diego, CA), 4-6 weeks old, were used in this study. All animal studies were conducted with an AntiCancer Institutional Animal Care and Use Committee (IACUC)-protocol specifically approved for this study and in accordance with the principals and procedures outlined in the National Institute of Health Guide for the Care and Use of Animals under Assurance Number A3873-1. In order to minimize any suffering of the animals, the use of anesthesia and analgesics were used for all surgical experiments. Animals were anesthetized by subcutaneous injection of a 0.02 ml solution of 20 mg/kg ketamine, 15.2 mg xylazine, and 0.48 mg/kg acepromazine maleate. The response of animals during surgery was monitored to ensure adequate depth of anesthesia. The animals were observed on a daily basis and humanely sacrificed by CO_2_ inhalation when they met the following humane endpoint criteria: severe tumor burden (more than 20 mm in diameter), prostration, significant body weight loss, difficulty breathing, rotational motion and body temperature drop. Animals were housed in a barrier facility on a high efficiency particulate arrestance (HEPA)-filtered rack under standard conditions of 12-hour light/dark cycles. The animals were fed an autoclaved laboratory rodent diet [25].

### Patient-derived tumor

A female patient diagnosed with a recurrent extranodal FDCS of the left lower extremity underwent surgical resection. She previously received adjuvant radiotherapy to the left lower extremity following resection of the primary tumor in 2014 and four cycles of chemotherapy with DOX and CTX for her recurrent disease. Chemotherapy was discontinued in April of 2015 due to medical co-morbidities and her inability to tolerate therapy. Surgical resection of the recurrent extra-nodal left lower extremity FDCS was performed by FCE on July 15, 2015. Written informed consent was obtained from the patient as part of a UCLA Institutional Review Board (IRB #10-001857)-approved protocol [25].

### Establishment of a PDOX model of FDCS by surgical orthotopic implantation (SOI)

A fresh sample of the FDCS of the patient was obtained and transported immediately to the laboratory at AntiCancer, Inc., on wet ice. The sample was cut into 5-mm fragments and implanted subcutaneously in nude mice. After three weeks, the subcutaneously-implanted tumors grew to more than 10 mm in diameter. The subcutaneously-grown tumors were then harvested and cut into small fragments (3 mm^3^). After nude mice were anesthetized with the ketamine solution described above, a 5-mm skin incision was made on the right high thigh into the biceps femoris, which was split to make space for the sarcoma tissue fragment. A single tumor fragment was implanted orthotopically into the space to establish the PDOX model. The wound was closed with a 6-0 nylon suture (Ethilon, Ethicon, Inc., NJ, USA) [25].

### Preparation and administration of *S. typhimurium* A1-R

*S. typhimurium* A1-R expressing GFP (AntiCancer, Inc., San Diego, CA, USA) was grown overnight on LB medium and then diluted 1:10 in LB medium. Bacteria were harvested at late-log phase, washed with PBS, and then diluted in PBS. *S. typhimurium*A1-R was injected intraperitoneally. A total of 2 × 10^7^ CFU *S. typhimurium* A1-R in 50 μl PBS was administered intraperitoneally (i.p.) in the follicular dendritic cell sarcoma-bearing mice [25].

### Treatment study design in the PDOX model of soft tissue sarcoma

PDOX mouse models were randomized into seven groups of five mice each (Figure 1): Group 1, control with PBS, i.p.; Group 2, treated with BEZ, 50 mg/kg, oral gavage, q5/W for 4 weeks; Group 3, treated with DOX, 2.4 mg/kg, i.p., qW for 4 weeks; Group 4, treated with DOX, 2.4 mg/kg, i.p., qW for 2 weeks; and BEZ, 50 mg/kg, oral gavage, q5/W for 2 weeks; Group 5, treated with *S. typhimurium* A1-R, 2.5 × 10^7^ CFU, i.p., qW for 4 weeks; Group 6, treated with *S. typhimurium* A1-R, 2.5 × 10^7^ CFU, i.p., qW for 2 weeks followed by DOX, 2.4 mg/kg, i.p., qW for 2 weeks; and Group 7, treated with *S. typhimurium* A1-R, 2.5 × 10^7^ CFU, i.p., qW for 2 weeks followed by BEZ, 50 mg/kg, oral gavage, q5/W for 2 weeks. Tumor length, width and mouse body weight were measured twice a week. Tumor volume was calculated with the following formula: Tumor volume (mm^3^) = length (mm) × width (mm) × width (mm) × 1/2. Data are presented as mean ± SD. The tumor valume ratio is defined at the tumor volume at any given time point relative to the initial tumor volume. All treated mice except Group 2 were sacrificed on day-29, and tumors were resected for further histological evaluation. Mice treated with BEZ were sacrificed on day-18 due to outgrowth of the tumors.

### Histological examination

Fresh tumor samples were fixed in 10% formalin and embedded in paraffin before sectioning and staining. Tissue sections (5 μm) were deparaffinized in xylene and rehydrated in an ethanol series. Hematoxylin and eosin (H&E) staining was performed according to standard protocol. Histological examination was performed with a BHS system microscope. Images were acquired with INFINITY ANALYZE software (Lumenera Corporation, Ottawa, Canada). Grade IV: no viable tumor is detectible [25].

### Imaging of tumor-targeted bacteria

The FV1000 confocal microscope (Olympus) [39] was used to image resected tumors for the presence of *S. typhimurium* A1-R-GFP. The OV100 (Olympus) variable-magnification fluorescence imager [40] was used to image colonies of *S. typhimurium* A1-R from resected tumors.

### Statistical analysis

SPSS statistics version 21.0 was used for all statistical analyses (IBM, New York City, NY, USA). Significant differences for continuous variables were determined using the Mann-Whitney U test. A probability value of *P* < 0.05 was considered statistically significant [25].
